# Supplementary material for: A Comprehensive Assessment of Ultraviolet-Radiation-Induced Mutations in Flammulina filiformis Using Whole-Genome Resequencing
Source: J Fungi (Basel). 2024 Mar 20;10(3):228. doi: 10.3390/jof10030228 (PMC10971301; doi:10.3390/jof10030228)
Supplement: Supplementary file 1 [file jof-10-00228-s001.zip › Supplementary Material S8/KEGG annotation/out/64381550635650.os/KO/out_map/map03460.html]

KEGG PATHWAY: Fanconi anemia pathway - Reference pathway


|  |  |
| --- | --- |
| **Fanconi anemia pathway - Reference pathway** |  |

[
Pathway menu
| Organism menu
| Pathway entry
| Show description
| User data mapping
]

The Fanconi anemia pathway is required for the efficient repair of damaged DNA, especially interstrand cross-links (ICLs). DNA ICL is directly recognized by FANCM and associated proteins, that recruit the FA core complex. The FA core complex monoubiquitinates FANCD2 and FANCI. The monoubiquitinated FANCD2/FANCI becomes an active form and interacts with a series of DNA repair proteins and facilitates downstream repair pathways. Fanconi anemia is caused by mutations in one of at least 13 FA genes and is characterized by congenital growth abnormalities, bone marrow failure and cancer predisposition.

|  |  |
| --- | --- |
| Reference pathway | 100% |
